# A mechanical method of cerebral cortical folding development based on thermal expansion

**DOI:** 10.1038/s41598-018-37461-2

**Published:** 2019-02-13

**Authors:** Linlin Wang, Jianyao Yao, Ning Hu

**Affiliations:** 1 College of Aerospace Engineering, Chongqing University, Chongqing, China; 2 Collaborative Innovation Center for Brain Science, Chongqing University, Chongqing, China; 3 Postdoctoral Station of Mechanics, Chongqing University, Chongqing, China

## Abstract

Cortical folding malformations are associated with several severe neurological disorders, including epilepsy, schizophrenia and autism. However, the mechanism behind cerebral cortical folding development is not yet clear. In this paper, we propose a mechanical method based on thermal expansion to simulate the development of human cerebral cortical folding. The influences of stiffness ratio, growth rate ratio, and initial cortical plate thickness on cortical folding are discussed. The results of our thermal expansion model are consistent with previous studies, indicating that abnormal values of the aforementioned three factors could directly lead to cortical folding malformation in a generally fixed pattern.

## Introduction

The complex anatomical structure of the cerebral cortex is directly related to complex brain functions. Despite being studied for a long time, the driving factors of cerebral morphology remain one of the most mysterious unanswered questions. A high degree of cortical folding may strongly correlate with human intellectual abilities^1,2^. Meanwhile, severe cortical folding malformations are thought to be associated with neurological disorders. For example, lissencephaly and polymicrogyria malformations can be accompanied by epilepsy^3^, schizophrenia^4^ or autism^5^. Discovering the evolutionary and developmental origins of cortical folding would help in understanding these abnormal cerebral cortical developments in neurodevelopmental disorders.

In the past decades, considerable efforts based on anatomy and cytology have been made to understand mammalian cerebral cortical folding. Many important anatomical phenomena and genetic factors have been found^1,6–12^. However, the ethical and technical limitations of experiments on primate species have restricted direct biological studies on cerebral cortical folding. It is difficult to explain the mechanism of cerebral cortical folding with cytological study alone. At the same time, the combination of mathematical/mechanical modelling and biomechanical experimental data provides a means to objectively and efficiently estimate the hypothesized mechanisms.

Historically, many theories about cerebral cortical folding have been proposed, such as the hypothesis that folding is the effect of cerebral cortical growth within the limits of the cranial volume^13^, and the influential view that the axonal tension produces folding by pulling on the cortex^14^. These hypotheses have all been proven to be inconsistent with the experimental evidence^15,16^. The growth-driven hypothesis is one of the most reasonable recent hypotheses, which assumes that the grey matter grows more quickly than the white matter. Then, the differential growth leads to a mechanical bulking that shapes the cortex.

Folding can be induced in the lissencephalic mouse brain by genetic manipulations that increase the cortical growth rate^11,17–21^. This biological experimental result is strong evidence for the growth-driven hypothesis. Moeskops *et al*.^22^ used the magnetic resonance imaging (MRI) method to study the development of cortical morphology. From 30 gestational week (GW) to 40 GW, the cortical grey matter volume increased by a factor of 4.6 and the subcortical white matter volume increased by 1.9^22^. This finding has successfully proved that the cortical grey matter grows more quickly than the white matter in gestation. Many studies depend on the growth-driven hypothesis^23–30^. Richman *et al*.^23^ used an analytical mechanical model to obtain the theoretical results of the cortical folding. Particularly, their model assumed that the outer and inner cortical layers, corresponding to grey matter and white matter, had different growth rates. Toro and Burnod^15^ simulated the cortical folding using an idealized geometrical model in which the cortical layer was a circle structure composed of quadrilateral elements which were attached to the centre of the model by line elements that represented the radial glia and axonal fibres. Dervaux *et al*.^24^ verified growth-driven hypothesis of biological growth by simulating the growth of simple thin hyperelastic samples. Two-dimensional models of cortical folding have studied the effects of cortical growth rate, cortical thickness, spatial variations, and mechanical feedback (Bayly *et al*.^25^), as well as stiffness ratio, cortical thickness and growth ratio on cortical folding (Budday *et al*.^26^) related to morphological abnormalities in the developing human brain (Budday *et al*.^27^). Ronan *et al*.^28^ adopted an MRI method to analyse the relationship between the intrinsic curvature of cortex and the degree of gyrification. Depending on their findings they concluded that differential expansion was a plausible primary mechanism for cortical folding. Tallinen *et al*.^29^ used both 2D and 3D models to simulate cortical folding, and a polydimethylsiloxane bilayer physical model was used to verify the numerical simulation results. Razavi *et al*.^30^ used a 3D model to analyse the critical growth ratios for instability in the brain model, the effects of cortical thickness and brain tissue material properties on cortical folding.

However, the real cortical folding is a sequence of complicated processes that starts from the growth of neuronal tubes, followed by neuronal proliferation, glial cell proliferation, neuronal migration and differentiation, axonal wiring, synaptogenesis and myelination^30^. On the other hand, the mechanical model only considers the mechanical factors in cortical folding.

Most of the studies based on the growth-driven hypothesis have adopted the finite growth theory in soft elastic tissues, which was first proposed by Rodriguez *et al*.^31^. The main ideas and basic equations of the finite growth theory are summarized below. During the growth process, the mass density ρ is supposed to be constant in time and position. Then, the mass conversation equation is simplified to

1 $$\frac{{\rm{dV}}}{{\rm{dt}}}={\rm{div}}v$$

where V is the tissue volume, t is the growth time, and *v* is the growth velocity vector. This equation indicates that when taking the mass density as constant, the tissue growth can be considered as the volume expansion. The real deformation of the tissue during growth is decomposed into two processes: (1) the tissue-independent growth deformation without elastic deformation, which may lead to geometric discontinuities such as holes and overlaps, and (2) the tissue elastic deformation, which occurs after the tissue-independent growth deformation, ensuring geometric continuity through the material constitutive equations. According to this assumption, the real deformation gradient tensor ***F*** is decomposed multiplicatively into the elastic deformation gradient tensor ***F***_***E***_ and the growth deformation gradient tensor ***F***_***G***_,

2 $$F={F}_{E}{F}_{G}$$

More details on the finite growth theory can be found in Rodriguez *et al*.^31^.

The main idea of the finite growth theory is quite similar to the thermal expansion theory. When taking the mass density ρ as constant, the tissue volume thermal expansion may be treated as tissue growth. Similarly, the real deformation through the thermal expansion process can also be decomposed into two independent processes: (1) the tissue independent thermal expansion deformation without elastic deformation, which may lead to geometric discontinuities such as holes and overlaps, and (2) the tissue elastic deformation, which occurs after the tissue independent thermal expansion deformation, ensuring the geometric continuity through the material constitutive equations. Then, the real deformation gradient tensor ***F*** is decomposed multiplicatively into the elastic deformation gradient tensor ***F***_***E***_ and the thermal expansion deformation gradient tensor ***F***_***TH***_,

3 $$F={F}_{E}{F}_{TH}$$

If we use the thermal expansion deformation tensor ***F***_***TH***_ to substitute the growth deformation tensor F_G_, then we can use the tissue thermal expansion process to simulate the tissue growth process. To the best of our knowledge, the qualitative analogy between growth and thermal expansion was first introduced by Skalak^32^. Similar opinions are also presented by Jones^33^, Roose^34^ and Volokh^35^. The experiments that use swelling gels to mimic the cerebral cortical folding can be partially considered as a proof of the thermal expansion model, because they both produce folding through the volume expansion^29,36,37^. Since there are many well-known commercial software packages that include powerful thermal expansion modelling functions, using the thermal expansion method to research the tissue growth process offers a simple, efficient approach. Razavi *et al*.^30^ have adopted a 3D model based on thermal expansion to analyse the role of mechanical factors in cortical folding, including the critical growth ratios for instability in the brain model, cortical thickness and brain tissue material properties effects on cortical folding. Their work is a successful example of using the thermal expansion method on cortical folding study. And their findings are also instructive to conduct our work.

In this thesis, we use the thermal expansion approach to research the mechanism of the cerebral cortical folding development. The model in this paper only simulates the 2-dimensional state and is mainly used to illustrate the basic trends in cortical folding, instead of accurately reflecting the expected behaviour in real brains. The mechanical module in the commercial software ANSYS is adopted to carry out the simulation. The simulation focuses on the three important factors, namely, brain tissue stiffness, growth rate and thickness. The reason for the cerebral cortical folding malformations will also be investigated. The importance of these aforementioned three factors has been reported by Razavi *et al*.^30^ and Budday *et al*.^26^, among others. The present study aims to replicate these basic behaviours using a simple, 2D model with thermal expansion.

## Mathematical Model and Simulation

The brain tissue growth rate could be defined as

4 $${\rm{G}}=\frac{{\rm{\Delta }}{\rm{L}}}{{\rm{L}}\cdot {\rm{\Delta }}{\rm{t}}}$$

where G is the brain tissue growth rate, L is the brain tissue length, ΔL is the brain tissue length increment by growth, and Δt is the brain tissue growth time.

The thermal expansion coefficient is defined as

5 $${\rm{\alpha }}=\frac{{\rm{\Delta }}{\rm{L}}}{{\rm{L}}\cdot {\rm{\Delta }}{\rm{\tau }}}$$

where α is the material thermal expansion coefficient, L is the material length, ΔL is the material length increment by thermal expansion, and Δτ is the temperature increment. If taking the temperature increment Δτ in Equation (5) as the growth time Δt in Equation (4), then the thermal expansion coefficient can simulate the brain tissue growth rate. In the following, the brain growth rate G denotes the thermal expansion coefficient α, and the growth time Δt denotes the temperature increment Δτ.

Brain tissue shows the time-dependent compressibility due to the poroelasticity^38^, but this is irrelevant over the long-term growth process. Therefore, we only considered the brain tissue elastic effects. In fact, the grey matter and white matter are both treated as hyperelastic materials in many reports^25–27,29,30,36,39^. In this paper, the Neo-Hooken hyperelastic material was used to model the grey matter and white matter. The strain energy function for the Neo-Hooken hyperelastic model is,

6 $$W=\frac{{\rm{\mu }}}{{\rm{2}}}[(\mathrm{Tr}(F{F}^{T}{)J}^{-2/3}-3)]+\frac{1}{d}{({\rm{J}}-1)}^{2}$$

where W is the strain energy per unit reference volume, μ is the material initial stain shear modulus, ***F*** is the deformation gradient tensor, J is the determinant of the deformation gradient tensor, and d is the material incompressibility parameter, which are defined as,

7 $${\rm{J}}=\det (F)=\det ({F}_{E}{F}_{TH})=\det ({F}_{E})\det ({F}_{TH})$$

and

8 $${\rm{d}}=\frac{2}{{\rm{k}}}$$

In Equation (8), k is the initial bulk modulus. μ and k can be obtained from the material Young’s modulus E and Poisson’s ratio ν.

Despite a huge number of *in vitro* or *in vivo* studies on brain biomechanics, it remains difficult to accurately characterize brain tissue^40^. Most of the MRE (magnetic resonance elastography) results show that the shear modulus of the white matter is 1.2–2.6 times higher than that of grey matter^40^. In contrast, other experimental results show that grey matter is stiffer than white matter^40,41^. The stiffness of the grey matter is even considered to be similar with that of the white matter^29,36,42^. As the absolute cortical stiffness is difficult to identify, we explored the role of the relative stiffness. We modelled the human cortical (grey matter) as Neo-Hooken elastic with Young’s modulus E_G_ = 9210.87 Pa and Poisson’s ratio ν = 0.458 (Soza^43^). The subcortical (white matter) was also modelled as Neo-Hooken elastic, whose Young’s modulus E_W_ will change depending on the stiffness ratio E_G_/E_W._

**Figure 1:**
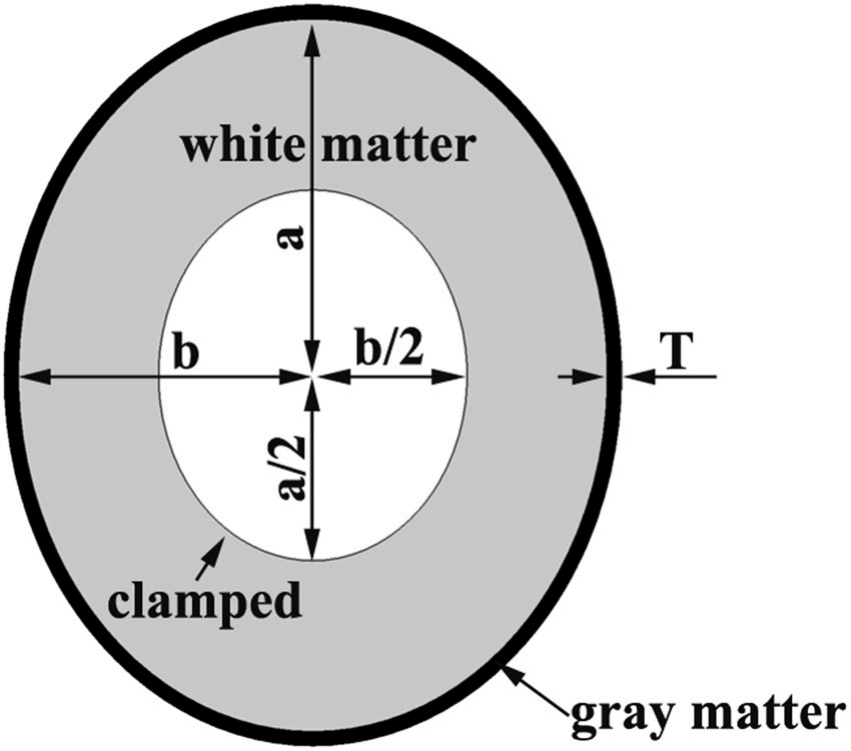
The simplified elliptic brain slice. The elliptic slice area was 40.69 cm^2^, with a/b = 1.2, a is the ellipse major axis radius, and b is the ellipse minor axis radius. The area weighted mean radius R was 36 mm. T denotes the cortical plate thickness. The thickness of the elliptic brain slice was T_SLICE_ = 0.01 mm. The inner core area of the elliptic brain slice was deleted. The deleted area was also an ellipse, whose major axis radius was a/2 and minor axis radius is b/2. When simulating, the inner boundary of the hollow elliptic brain slice was clamped.

The human transverse brain section (horizontal section) looks like an ellipse; therefore, we used an elliptic slice to approximately model the transverse brain section. We fixed the elliptic slice area to 40.69 cm^2^, with a/b = 1.2 (Fig. 1, a is the ellipse major axis radius, and b is the ellipse minor axis radius). The area weighted mean radius R was 36 mm. The thickness of the elliptic brain slice was T_SLICE_ = 0.01 mm. For such geometries, the resulting folding patterns are independent of the core area^29,30^; therefore the inner core area of the elliptic brain slice was deleted to save computational resources. The deleted area was also an ellipse whose major axis radius was a/2 and minor axis radius was b/2 (Fig. 1). The inner boundary of the hollow elliptic brain slice was clamped during simulation, similar to Tallinen *et al*.^29^.

The cortical plate thickness at 22 GW is approximately 1–1.5 mm^36^; thus we took the cortical plate thickness T = 1.5 mm as the standard initial cortical plate thickness in the simulation. When analysing the effect of the initial cortical plate thickness on the cortical folding, we generally changed the initial cortical plate thickness T in a few steps, based on T = 1.5 mm.

We mainly investigated the role of the relative growth rate. Fixing the grey matter growth rate G_G_, the white matter growth rate G_W_ will change depending on the growth rate ratio G_G_/G_W_. We fixed G_G_ = 0.002 and the growth time Δt = 500, which leads to an approximately 1-fold increment in tissue length (if ignoring the tissue elasticity). From the 22 GW to adulthood, the human brain volume can approximately increase from 60 ml to 1200 ml^36^, a 19-fold enlargement in brain volume, corresponding to a 1.71-fold increase in tissue length. Depending on Zhang^44^, in mammals, the brain grey matter volume V_G_ and white matter volume V_W_ have a robust empirical power law relation between them as

9 $${{\rm{V}}}_{{\rm{W}}}\, \sim \,{{\rm{V}}}_{G}^{1.23\pm 0.002}$$

The power law is derived from a large quantity of mammalian brain volume statistical data. Using the hollow elliptic brain slice (Fig. 1), the white matter volume can be calculated as

10 $${{\rm{V}}}_{{\rm{W}}}={({\rm{1}}+{{\rm{G}}}_{{\rm{W}}}\cdot {\rm{\Delta }}{\rm{t}})}^{{\rm{3}}}[{\rm{\pi }}({\rm{a}}-{\rm{T}})({\rm{b}}-{\rm{T}})-\frac{{\rm{1}}}{{\rm{4}}}{\rm{\pi }}\mathrm{ab}]{{\rm{T}}}_{{\rm{SLICE}}}$$

and the grey matter volume is

11 $${{\rm{V}}}_{{\rm{G}}}={({\rm{1}}+{{\rm{G}}}_{G}\cdot {\rm{\Delta }}{\rm{t}})}^{{\rm{3}}}[{\rm{\pi }}\mathrm{ab}-{\rm{\pi }}({\rm{a}}-{\rm{T}})({\rm{b}}-{\rm{T}})]{{\rm{T}}}_{{\rm{SLICE}}}$$

Assuming the volume of this very thin brain slice in our model still satisfies the above empirical scaling law, when the cortical plate thickness was the standard value T = 1.5 mm, we could obtain the growth rate ratio G_G_/G_W_ = 3.6 as the standard growth rate ratio in the following simulations.

Garcia *et al*.^45^ and Moeskops *et al*.^22^ used the MRI method to obtain the surface area and volume increase of the cortical grey matter and subcortical white matter. Their studies were carried out on preterm infants. Using the data (from 30 GW to 40 GW) in Moeskops *et al*.^22^, if we take the grey matter growth and white matter growth as isotropic growth, the growth rate ratio was approximately G_G_/G_W_ = 2.8. The G_G_/G_W_ = 3.6 estimated in this paper was close to the value G_G_/G_W_ = 2.8 calculated from Moeskops *et al*.^22^.

## Results

The simulations mainly focus on the effects of the stiffness ratio, the growth ratio and the initial cortical plate thickness on cortical folding development, especially on the cortical folding malformations such as pachygyria, lissencephaly and polymicrogyria. The detailed simulations are as follows.

### The effect of the stiffness ratio

To explore the effect of the stiffness ratio E_G_/E_W_, we used the hollow elliptic slice brain section model in Fig. 1. The initial cortical plate thickness was fixed to 1.5 mm. The growth rate ratio was G_G_/G_W_ = 3.6. We discretized this hollow elliptic slice with 7344 3-node triangle elements and assumed a plane strain state^26,29^. The boundary of the cortical circumference had 216 nodes. In each case, the grey matter layer contained at least six layers through its thickness.

We fixed the grey matter Young’s modulus E_**G**_ = 9210.87 Pa and Poisson’s ratio ν = 0.458, and the stiffness ratio E_G_/E_W_ varied by changing the white matter Young’s modulus E_W_. The white matter Poisson’s ratio was also fixed to ν = 0.458. Although the absolute stiffness of the brain tissue varies widely, the maximum stiffness ratio between white matter and grey matter has never been reported to exceed 3. In this paper, the stiffness ratio E_G_/E_W_ adopted these values: 1/3, 1/2.61 (Kruse^46^), 1/2, 1/1.36 (Budday^47^), 1 (Tallinen^29,36^, Shuck^42^), 1.54 (Christ^48^), 2 and 3.

**Figure 2:**
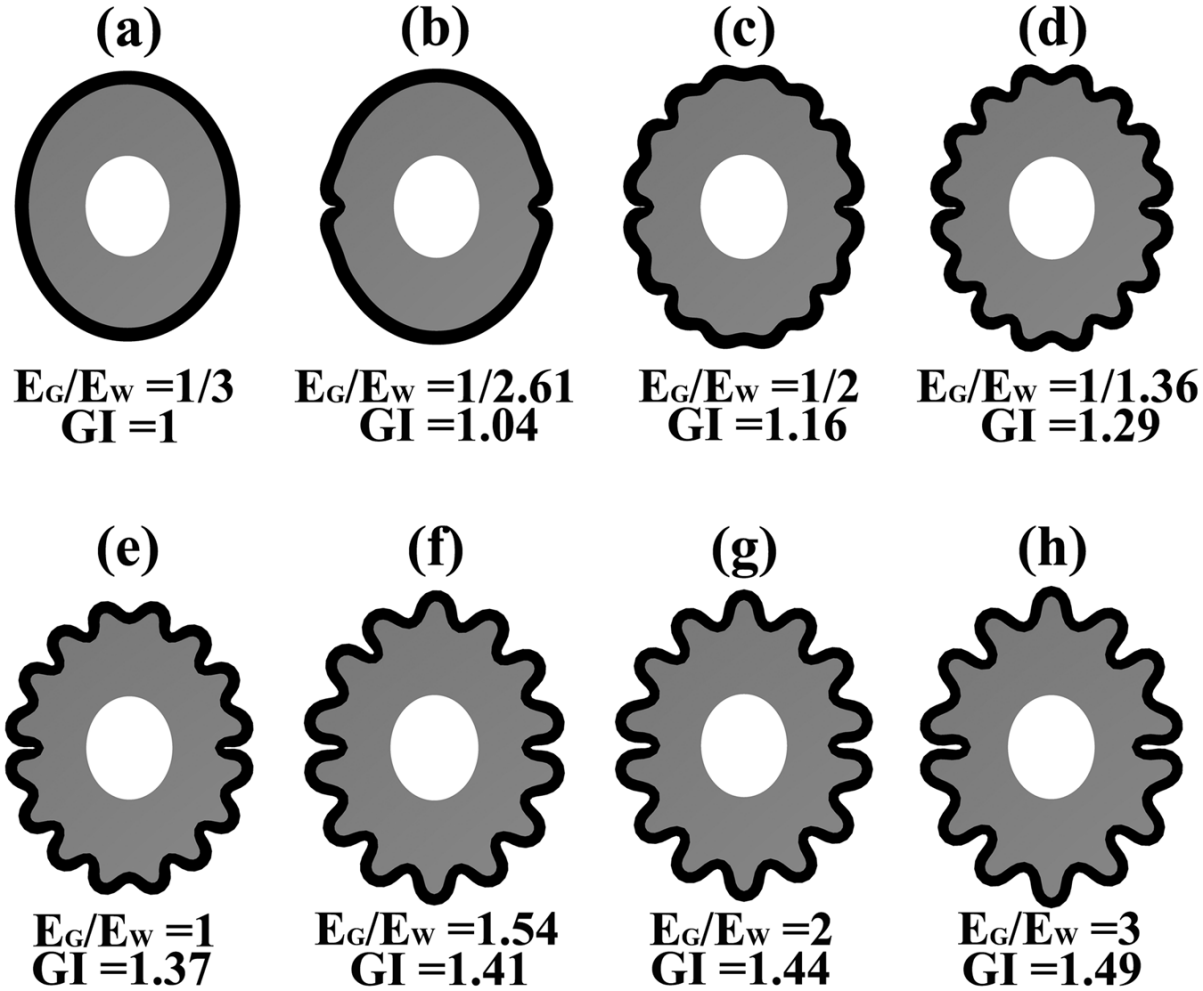
The effect of the stiffness ratio E_G_/E_W_. From (**a**–**h**), the stiffness ratio increased from E_G_/E_W_ = 1/3 to E_G_/E_W_ = 3. From (**a**–**h**), the GI (gyrification index) increased with the stiffness ratio E_G_/E_W_.

Figure 2 illustrates the sensitivity of the cortical folding pattern with respect to the stiffness ratio E_G_/E_W_. The stiffness ratio E_G_/E_W_ and cortex gyrification index (GI)^49^ are displayed in the figure. GI is a measure of the degree of folding and is defined as the ratio between the total outer cortex surface and the superficially exposed part of the outer surface. The stiffness ratio E_G_/E_W_ has a great influence on cortical folding pattern. From Fig. 2(a–h), the GI increased with the stiffness ratio E_G_/E_W_.

**Figure 3:**
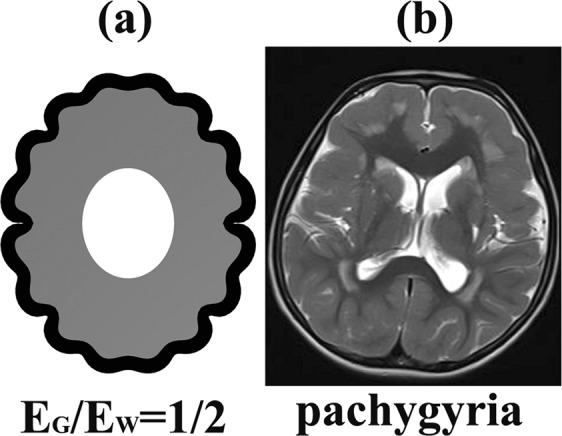
The pachygyria. (**a**) The cortical folding simulation result at stiffness ratio E_G_/E_W_ = 1/2. (**b**) The MRI (magnetic resonance imaging) of pachygyria. Case courtesy of Dr. Vinay Shah, Radiopaedia.org, rlD: 20767. When the stiffness ratio E_G_/E_W_ = 1/2, the sulci became very shallow. The neighbouring gyri merged into each other forming a huge and flat gyrus, which was very similar to the pachygyria.

**Figure 4:**
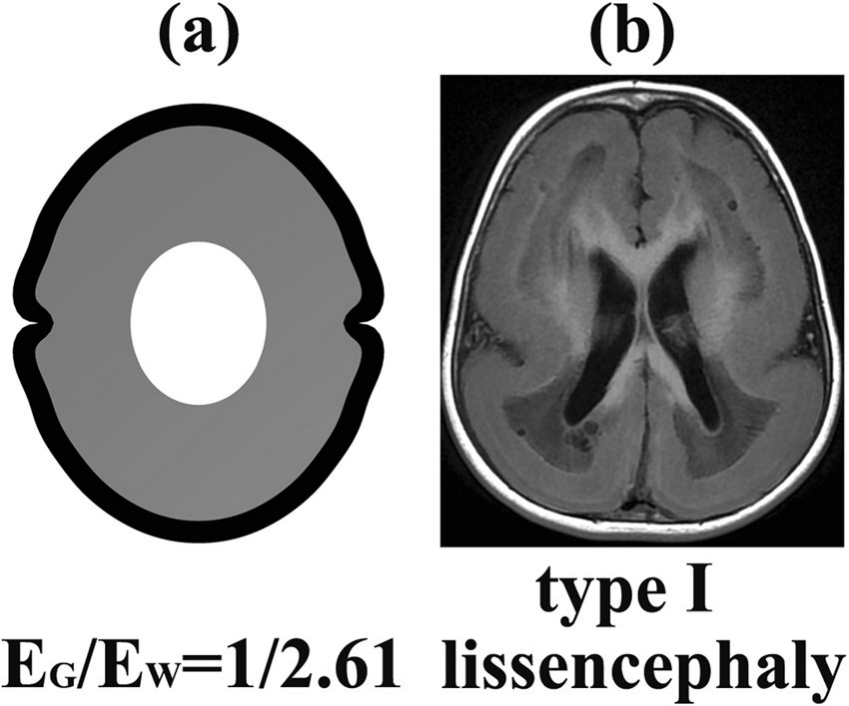
The type І lissencephaly. (**a**) The cortical folding simulation result at stiffness ratio E_G_/E_W_ = 1/2.61. (**b**) The MRI of type І lissencephaly. Case courtesy of Dr. Amro Omar, Radiopaedia.org, rlD: 3628. When the stiffness ratio decreased to E_G_/E_W_ = 1/2.61, the cerebral cortical folding was type І lissencephaly.

When the stiffness ratio E_G_/E_W_ ≤ 1/2, the cerebral cortical folding patterns were abnormal (Fig. 2(a–c)). When the stiffness ratio E_G_/E_W_ = 1/2, the sulci became very shallow. The neighbouring gyri merged into each other, forming a huge and flat gyrus, which is very similar to pachygyria (Fig. 3). When the stiffness ratio decreased to E_G_/E_W_ = 1/2.61, the cerebral cortical folding showed a type І lissencephaly^50,51^ (Fig. 4). When the stiffness ratio decreased to E_G_/E_W_ = 1/3, the cerebral cortical folding remained totally smooth (Fig. 2(a)). The relationship between the brain tissue stiffness ratio and cerebral cortical folding malformation can be successfully established using the proposed method.

The results in Figs 3 and 4 are very similar to the cerebral cortical folding malformation anatomical features. It is clear that the cerebral cortical folding may become pachygyria and lissencephaly when the stiffness ratio E_G_/E_W_ is small enough. Thus far, reported, the minimum stiffness ratio E_G_/E_W_ in the normal human brain is E_G_/E_W_ = 1/2.61 (Chatelin^40^, Kruse^46^).

It should be noted that infections and lesions may lead to changes in brain tissue stiffness, and further development of malformed cortical folding. However, the biomechanical data of the malformed brain tissues is rarely reported, especially in developing brains. Therefore, the brain tissue stiffness experiments should also be carried out on malformed brain tissues.

When the stiffness ratio increases from E_G_/E_W_ < 1 to E_G_/E_W_ > 1, the folding may experience three different mechanical status^29^: (1) a soft layer of grey matter grows on a stiff white matter substrate (E_G_/E_W_ < 1), (2) a layer of grey matter grows on a white matter substrate with identical stiffness (E_G_/E_W_ = 1), and (3) a stiff layer of grey matter grows on a soft white matter substrate (E_G_/E_W_ > 1).

When the stiffness ratio E_G_/E_W_ > 1, the simulation results were similar to the typical mechanical wrinkling, where a stiff outer layer grows on a soft substrate. This type of wrinkling may lead to sinusoidal folding, where both the gyri and sulci are smooth^29^. The same folding patterns were also observed in the numerical results as shown from Fig. 2(f–h), where the cortex layer looked like a sinusoidal layer with consistent thickness. This phenomenon became more pronounced when the grey matter was more rigid. This finding is consistent with Tallienen *et al*.^29^.

When the grey matter was not stiffer than the white matter (E_G_/E_W_ ≤ 1), the sulci became more cusped, and the cortex at sulci fundi became thinner than that at the gyri crowns. This finding is similar to Tallienen *et al*.^29^. In the real brain, most of the real brain sulci have a cusped shape, the cortex thickness is not identical, and the thickness at gyri crowns is greater than that at the sulci fundi^29^. When grey matter was stiffer than white matter (E_G_/E_W_ > 1), the grey matter layer looked like a sinusoidal layer with consistent thickness, which is not similar to the real brain cortex. To the best of our knowledge, there are few investigations supporting the hypothesis that grey matter is stiffer than white matter.

### The effect of the growth rate ratio

To investigate the effect of the growth rate ratio on cortical folding, we used the same hollow elliptic slice (Fig. 1). The initial cortical plate thickness was fixed to 1.5 mm. The mesh discrete method was as the same as in the previous section. The stiffness ratio E_G_/E_W_ adopted two values in this part, E_G_/E_W_ = 1/1.36 and E_G_/E_W_ = 1. Taking G_G_/G_W_ = 3.6 as the standard value, the growth rate ratio was drastically changed to verify its effect on cortical folding. We fixed G_G_ = 0.002 and changed G_W_ to obtain different growth rate ratios. The growth rate ratio took G_G_/G_W_ = 1, G_G_/G_W_ = 3.6, G_G_/G_W_ = 36 and G_G_/G_W_ = 360. Additionally, the threshold of G_G_/G_W_ that induces obvious cortical folding was also investigated.

**Figure 5:**
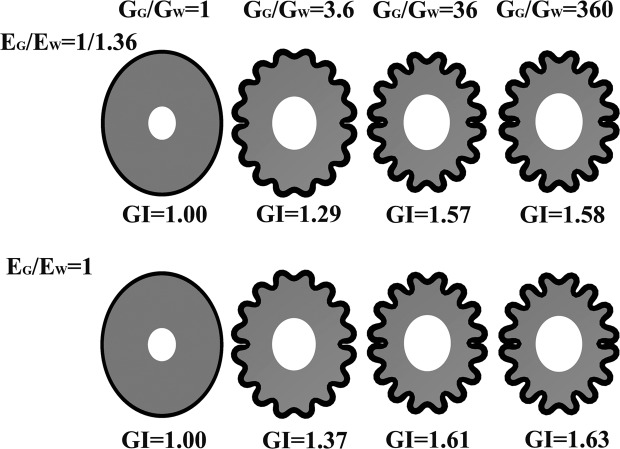
The effect of growth rate ratio. From left to right, the growth rate ratio varied from G_G_/G_W_ = 1 to G_G_/G_W_ = 360. The top row results had the same stiffness ratio E_G_/E_W_ = 1/1.36. The bottom row results had the same stiffness ratio E_G_/E_W_ = 1. When the growth rate ratio G_G_/G_W_ increased, the GI increased, but the GI increase rate decreased. When the growth rate of the white matter was as same as the grey matter (G_G_/G_W_ = 1), the cortex remained totally smooth. When the growth rate ratio increased from the normal value G_G_/G_W_ = 3.6 to G_G_/G_W_ = 360, the sulci became deeper and deeper, increasing the GI, but no malformation occured.

In Fig. 5, the results shown in the first row were obtained with the same stiffness ratio E_G_/E_W_ = 1/1.36, while the ones shown in the second row were obtained with the same stiffness ratio E_G_/E_W_ = 1. The images are scaled to fit the figure size to depict the shape of the cortical folding rather than the volume of the brain tissue. The results indicate that when the growth rate ratio G_G_/G_W_ increased, the GI increased, but the GI increase rate decreased. When the growth rate of the white matter was as same as the grey matter (G_G_/G_W_ = 1), the cortex remained totally smooth. When the growth rate ratio increased from the standard value G_G_/G_W_ = 3.6 to G_G_/G_W_ = 360, the sulci became deeper and GI also increased, but no malformation appeared.

When E_G_/E_W_ = 1, the threshold of the growth rate ratio that induced obvious cortical folding was G_G_/G_W_ = 1.71 (See Supplementary Fig. S1). When E_G_/E_W_ = 1/1.36, the threshold of the growth ratio that induced obvious cortical folding was G_G_/G_W_ = 2.02 (See Supplementary Fig. S2). In Supplementary Figs S1 and S2, it is clear that when the growth rate ratio increased from G_G_/G_W_ = 1 to the standard value G_G_/G_W_ = 3.6, the cortical folding pattern gradually experienced four states: totally smooth, lissencephaly, pachygyria and the normal state. It might be concluded that when the growth rate ratio G_G_/G_W_ was sufficiently small, cortical folding malformation may occur.

### The effect of the initial cortical plate thickness

To explore the effect of the initial cortical plate thickness on cortical folding, we used the hollow elliptic slice in Fig. 1. First, the initial cortical plate thickness adopted three values: T = 0.75 mm, T = 1.5 mm and T = 3 mm. After that, we gradually changed the cortex thickness to identify at which thickness the folding became lissencephaly, and only that result was recorded. The mesh discrete method was as the same as in the previous part. In this part, the stiffness ratio adopted was E_G_/E_W_ = 1/1.36 and E_G_/E_W_ = 1. The growth rate ratio was G_G_/G_W_ = 3.6.

**Figure 6:**
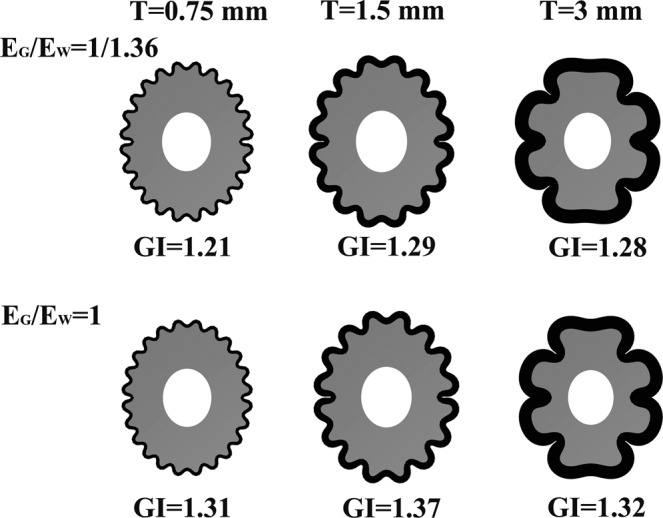
The effect of initial cortical plate thickness. The top row results had the same stiffness ratio E_G_/E_W_ = 1/1.36. The bottom row results had the same stiffness ratio E_G_/E_W_ = 1. From left to right the initial cortical plate thickness increased from 0.75 mm to 3 mm. When the initial cortical plate thickness was the standard thickness T = 1.5 mm, the cortex had the maximum GI (GI = 1.29, when E_G_/E_W_ = 1/1.36. GI = 1.37, when E_G_/E_W_ = 1). When the initial cortical plate thickness decreased from T = 1.5 mm to T = 0.75 mm, the number of gyrus and sulci increased, but the sulci depth decreased, finally the GI decreased. With numerous small gyri and shallow sulci, the folding pattern at T = 0.75 mm was typical polymicrogyria. When the initial cortical plate thickness increased to 3 mm, the gyri became bigger and the sulci became deeper, but the total number of gyri and sulci decreased, finally the GI decreased. The cortical folding pattern at this thickness resembled pachygyria.

In Fig. 6, the results shown in the first row were obtained using the same stiffness ratio E_G_/E_W_ = 1/1.36, and the results in the second row were obtained with the same stiffness ratio E_G_/E_W_ = 1. From left to right, the initial cortical plate thickness increased from 0.75 mm to 3 mm. For both investigated stiffness ratios, similar cortical folding development patterns were observed with the increase of cortical plate thickness. When the initial cortical plate thickness was the standard thickness T = 1.5 mm, the cortex had the maximum GI (GI = 1.29, when E_G_/E_W_ = 1/1.36. GI = 1.37, when E_G_/E_W_ = 1).

**Figure 7:**
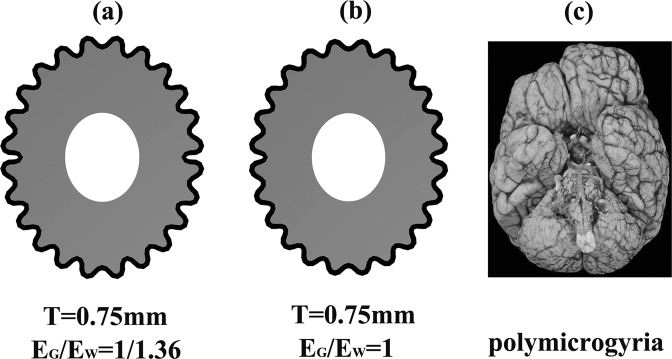
The polymicrogyria. (**a**) The cortical folding at stiffness ratio E_G_/E_W_ = 1/1.36 and initial cortical thickness T = 0.75 mm. (**b**) The cortical folding at stiffness ratio E_G_/E_W_ = 1 and initial cortical thickness T = 0.75 mm. (**c**) The polymicrogyria brain. Case courtesy of A. Prof Frank Gaillard, Radiopaedia.org, rID: 27813. With numerous small gyri and shallow sulci, the folding pattern at T = 0.75 mm was typical polymicrogyria. The folding pattern in (**a**,**b**) was quite similar to the polymicrogyria brain (**c**).

When the initial cortical plate thickness decreased from T = 1.5 mm to T = 0.75 mm, the number of gyrus and sulci increased, but the sulci depth decreased, and finally led to the decrease of GI. With numerous small gyri and shallow sulci, the folding pattern at T = 0.75 mm was typical polymicrogyria^52^. As mentioned above, the foetal cortical plate thickness at 22 GW is 1 mm-1.5 mm; thus, T = 0.75 mm is below the normal thickness range. The results indicate that when the cortical plate thickness is below the normal range, cortical folding may become to polymicrogyria, which is in accordance with Judkins^52^. In Judkins’ research, there is a reduction in the thickness of polymicrogyria cortex. Our simulations accurately show the thickness effect on polymicrogyria. Figure 7 shows the similarity between the simulation results and the polymicrogyria brain.

When the initial cortical plate thickness increased to 3 mm, whether E_G_/E_W_ = 1/1.36 or E_G_/E_W_ = 1, the gyri became bigger and the sulci became deeper, but the total number of gyri and sulci decreased; finally, the GI decreased. The cortical folding pattern at this thickness resembled pachygyria.

**Figure 8:**
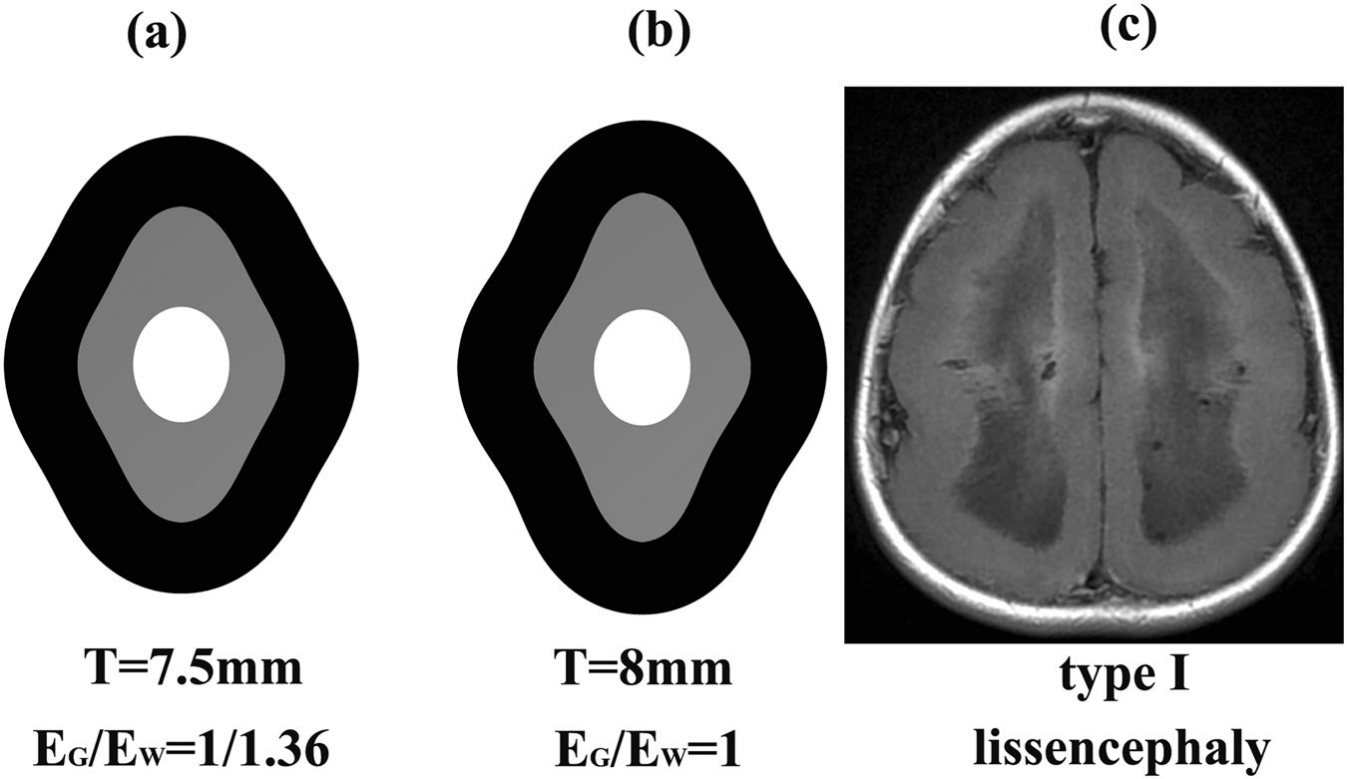
The lissencephaly. (**a**) The cortical folding at stiffness ratio E_G_/E_W_ = 1/1.36 and initial cortical thickness T = 7.5 mm. (**b**) The cortical folding at stiffness ratio E_G_/E_W_ = 1 and initial cortical thickness T = 8 mm. (**c**) The MRI of type І lissencephaly. Case courtesy of Dr. Amro Omar, Radiopaedia.org, rlD: 32628. When the thickness was 7.5 mm (E_G_/E_W_ = 1/1.36) and 8 mm (E_G_/E_W_ = 1), the cortical folding became type І lissencephaly. The stiffer the white matter, the more easily the lissencephaly appears.

Further increasing the initial cortical plate thickness, the gyri and sulci number continuously decreased. As shown in Fig. 8, when the thicknesses were 7.5 mm (E_G_/E_W_ = 1/1.36) and 8 mm (E_G_/E_W_ = 1), the cortical folding became type І lissencephaly. This finding indicates that when the initial cortical plate thickness is far above the normal thickness (1 mm-1.5 mm at 22 GW), the cortical folding becomes lissencephaly, and the smaller the E_G_/E_W_, the more easily lissencephaly appears. Firth^50^ showed that the cortical thickness may increase significantly in type І lissencephaly, usually varying from 15 mm to 20 mm, which is consistent with our result. As mentioned above, the mean brain radius R in the investigated model was 36 mm. When the initial cortical thickness was 1.5 mm, the radius-to-thickness ratio R/T = 24; when the initial cortical thickness was 7.5 mm and 8 mm, the radius-to-thickness ratio R/T ≤ 4. Therefore, it could be concluded that when the radius-to-thickness ratio is small enough, the cerebral cortical folding may become lissencephaly.

## Discussion

The simple mechanical model based on the thermal expansion method has successfully simulated the development of human cerebral cortical folding. The effect of the stiffness ratio E_G_/E_W_, the growth ratio G_G_/G_W_ and the initial cortical plate thickness on cortical folding are investigated in detail.

The stiffness ratio E_G_/ E_W_ has a significant effect on the cortical folding pattern. When the stiffness ratio E_G_/E_W_ is small enough (in this paper, when E_G_/E_W_ ≤ 1/2, G_G_/G_W_ = 3.6), cortical folding will become pachygyria (E_G_/E_W_ = 1/2, G_G_/G_W_ = 3.6) or lissencephaly (E_G_/E_W_ = 1/2.61, G_G_/G_W_ = 3.6). When the stiffness ratio increased to 1/2 < E_G_/E_W_ ≤ 1, the proposed method captured typical human cerebral cortical folding features such as the cusped sulci, the smooth gyri, the thickened gyri crowns and the thinned fundi. When the stiffness ratio E_G_/E_W_ was large enough (in this paper, E_G_/E_W_ > 1), the grey matter layer (cortex) looked like a sinusoidal layer with consistent thickness, which is not similar to the real brain cortex. The importance of the stiffness ratio on cortical folding found in this paper is similar to Budday *et al*.^26^ (2D model), Tallinen *et al*.^29^ (2D model) and Razavi *et al*.^30^ (3D model).

The growth ratio G_G_/G_W_ also has an obvious influence on cortical folding. When the growth ratio G_G_/G_W_ increased from the reasonable value G_G_/G_W_ = 3.6 to G_G_/G_W_ = 360, only the cerebral cortex GI increased, but no malformation occurred. When the growth rate ratio G_G_/G_W_ is small enough, the cortical folding malformation may occur.

The initial cortical plate thickness has an important effect on cortical folding as well. Polymicrogyria is mainly caused by the initial cortical plate thickness decrease. When the initial cortical plate thickness is smaller than the reasonable value (1 mm-1.5 mm at 22 GW), the cortical folding may become polymicrogyria. As the initial cortical plate thickness increases, the radius-to-thickness ratio R/T decreases. When the radius-to-thickness ratio R/T is small enough (in this paper, when R/T ≤ 4, G_G_/G_W_ = 3.6), the cortical folding may become lissencephaly. This finding is consistent with Budday *et al*.^26^ (2D model), Tallinen *et al*.^29^ (3D model) and Razavi *et al*.^30^ (3D model).

The consistency between the numerical results and the biological observations indicate that the thermal expansion method used in this paper is useful to illustrate the basic trends in cerebral cortical folding. The model used in this paper only considered the 2D state and adopted the isotropy and homogeneity assumption. All these limitations make the model in this paper unsuitable for precisely capturing the behaviour in real cerebral cortical folding. The model based on the thermal expansion method should be further improved in future research.

## Supplementary information

A mechanical method of cerebral cortical folding development based on thermal expansion

A mechanical method of cerebral cortical folding development based on thermal expansion-with tracked changes

## Data Availability

No datasets were generated or analysed during the current study.
