# Supplementary material for: A mechanical method of cerebral cortical folding development based on thermal expansion
Source: Sci Rep. 2019 Feb 13;9:1914. doi: 10.1038/s41598-018-37461-2 (PMC6374467; doi:10.1038/s41598-018-37461-2)
Supplement: Supplementary file 1 — A mechanical method of cerebral cortical folding development based on thermal expansion [file 41598_2018_37461_MOESM1_ESM.pdf]

A mechanical method of cerebral cortical folding development based on thermal expansion

Linlin Wang[1,2,3], Jianyao Yao[*1,2], Ning Hu[1,2]

[1] College of Aerospace Engineering, Chongqing University

[2] Collaborative Innovation Center for Brain Science, Chongqing University

[3] Postdoctoral Station of Mechanics, Chongqing University

[*]No.174, Shazheng Street, Shapingba District, Chongqing, China

*Telephone number: 86-23-65102510

*Facsimile number: 86-23-65102510

*Email: yaojianyao@cqu.edu.cn

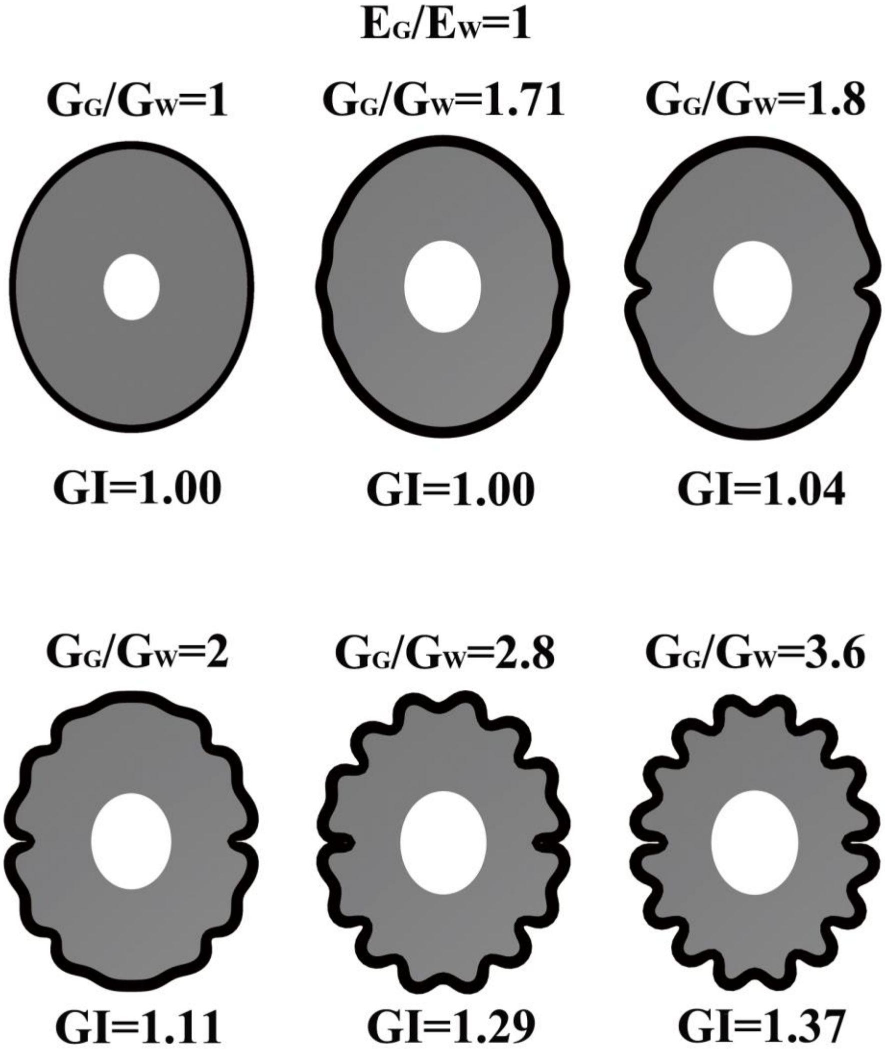

Figure S1 │The effect of growth rate ratio from G_G/G_W =1 to G_G/G_W =3.6 when E_G/E_W=1. The threshold of the growth rate ratio that induced obvious cortical folding was G_G/G_W=1.71. When the growth rate ratio increasd from G_G/G_W=1 to the standard value G_G/G_W=3.6, the cortical folding pattern gradually experienced four states, totally smooth, lissencephaly, pachygyria and the normal state. The images were scaled to fit the figure size to depict the shape of the cortical folding rather than the volume of the brain tissue.

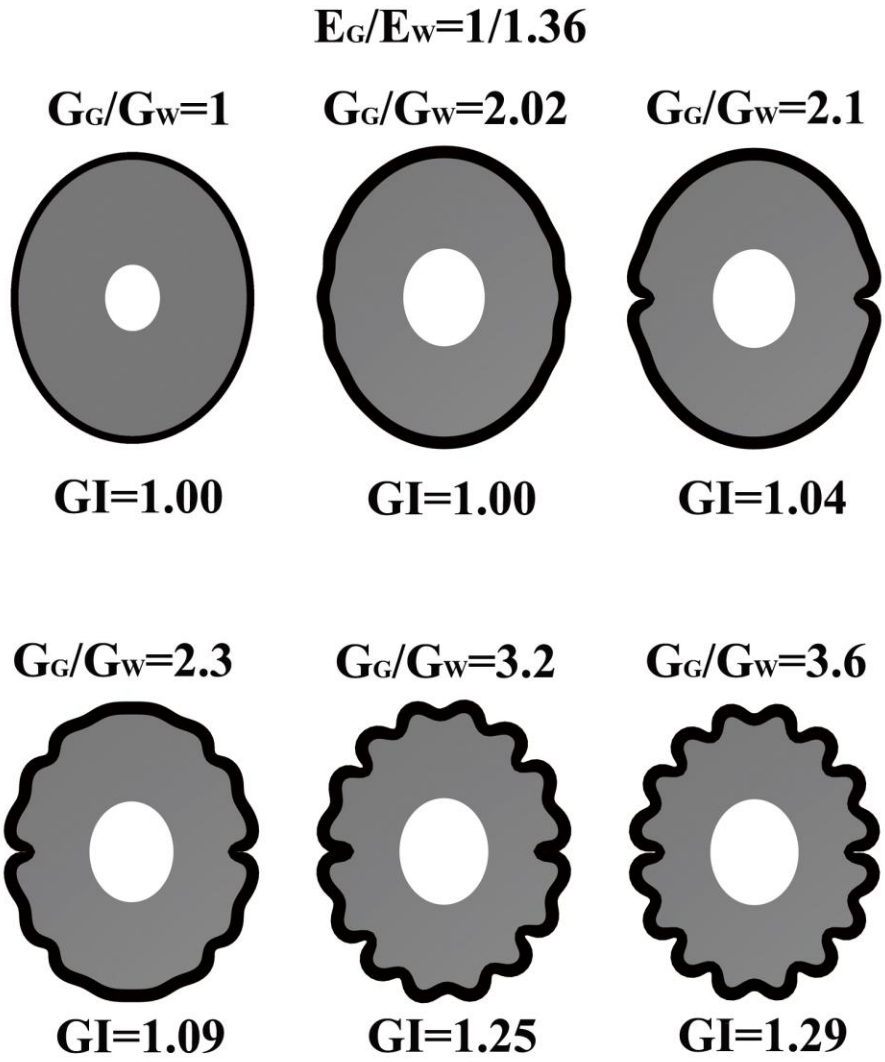

Figure S2 │The effect of growth rate ratio from G_G/G_W =1 to G_G/G_W =3.6 when E_G/E_W=1/1.36. The threshold of the growth rate ratio that induced obvious cortical folding was G_G/G_W=2.02. When the growth rate ratio increased from G_G/G_W=1 to the standard value G_G/G_W=3.6, the cortical folding pattern gradually experienced four states, totally smooth, lissencephaly, pachygyria and the normal state. The images were scaled to fit the figure size to depict the shape of the cortical folding rather than the volume of the brain tissue.
